# Application of Terahertz Technology in Food Safety: Rice Origin–Variety Classification Based on Spectral Analysis and Machine Learning

**DOI:** 10.3390/foods15111984

**Published:** 2026-06-03

**Authors:** Dongdong Ye, Xiaochang Yuan, Jianfei Xu, Chengjun Wang, Longhai Liu, Houli Liu, Jiabao Li, Depeng Ren, Chunlin Li

**Affiliations:** 1 School of Artificial Intelligence, Anhui Polytechnic University, Wuhu 241000, China; ddyecust@ahpu.edu.cn (D.Y.); 2230142161@stu.ahpu.edu.cn (X.Y.); cumt1279@163.com (C.W.); hlliu@ahpu.edu.cn (H.L.); lijiabao@ahpu.edu.cn (J.L.); 3221902110@stu.ahpu.edu.cn (D.R.); 2 Aviation Industry Corporation Huadong Photoelectric Co., Ltd., Wuhu 241003, China; 3 School of Intelligent Manufacturing, Wuhu University, Wuhu 241008, China; 4 Key Laboratory of Opto-Electronics Information Technology, Ministry of Education, School of Precision Instruments and Opto-Electronics Engineering, Tianjin University, Tianjin 300072, China; liulonghai@tju.edu.cn; 5 Key Laboratory of Information Traceability for Agricultural Products, Ministry of Agriculture and Rural Affairs, Zhejiang Academy of Agricultural Sciences, Hangzhou 310021, China; 6 State Key Laboratory for Quality and Safety of Agro-Products, Institute of Agro-Products Safety and Nutrition, Zhejiang Academy of Agricultural Sciences, Hangzhou 310021, China

**Keywords:** terahertz time-domain spectroscopy, rice, origin category, machine learning

## Abstract

Food security serves as a vital cornerstone for social stability. As one of the most important staple crops globally, the quality and geographical origin of rice are directly associated with consumer health. Traditional methods for classifying rice by origin and variety rely on sensory evaluation and manual inspection, which are subject to uncertainty and human error. To address this, this paper proposes a method for classifying rice by origin and variety based on terahertz time-domain spectroscopy. Terahertz technology features the advantages of non-destructive, high-sensitivity and non-contact detection, making it well-suited for food detection. This study employs terahertz time-domain spectroscopy combined with machine learning modeling methods, using 20 types of rice as the subject of investigation, with a focus on modeling and analyzing four representative samples. Refractive index and absorption coefficient were extracted through preprocessing methods including Savitzky–Golay convolution smoothing, wavelet denoising and moving average smoothing. Modeling, classification, and detection were implemented using principal component analysis, partial least squares discriminant analysis, and least-squares support vector machine. The experimental results indicate that principal component analysis (PCA) alone performs poorly in classification tasks. However, a classification model combining PCA for dimensionality reduction with a least-squares support vector machine (SVM), following Savitzky–Golay smoothing, demonstrated the best performance, achieving a prediction accuracy of 93.3%. In an extended test involving 20 samples, the model achieved an identification accuracy of 89.6%. Quantitative metrics demonstrate the feasibility of using terahertz technology combined with optimized machine learning algorithms for classifying rice by origin and variety.

## 1. Introduction

Food security is closely related to public health and social stability, and has long been a key focus in food science research. As one of the most important staple crops worldwide, rice plays an irreplaceable role in the daily diet. However, annual post-harvest losses of grains remain quite significant, further underscoring the importance of classifying and identifying grain origins and varieties [1]. Traditional methods for classifying rice by origin and variety primarily rely on sensory quality—including appearance, aroma, and texture—as well as label tracking, paper-based documentation, and manual verification [2,3,4]. However, these methods have certain drawbacks. Relying solely on texture to determine rice origin also carries a degree of uncertainty. Furthermore, traditional methods for classifying rice by origin and variety are susceptible to human interference. For example, unscrupulous merchants may add chemicals or alter processing methods to conceal the true origin of the rice, thereby misleading consumers. Therefore, researching and validating a more sensitive and reliable method for classifying rice by origin and variety holds significant scientific importance.

In recent years, spectroscopic and chromatographic techniques have been gradually applied to grain detection and quality evaluation. Wang Huixin et al. used near-infrared spectroscopy combined with machine learning to achieve quantitative analysis of moisture, protein, and amylose in rice, supporting rice quality identification [5]. Zhang Da et al. established an ultrasonic-assisted liquid chromatography method for rapid detection of fluconazole residues in rice with improved efficiency [6]. Yu Yunxin et al. applied hyperspectral technology combined with multivariate analysis to realize accurate classification of adulterated rice [7]. Although these methods have made certain progress, some still have limitations in detection speed, operation complexity, or non-destructiveness in practical application.

Terahertz time-domain spectroscopy (THz-TDS) is an emerging non-destructive analytical technique with high sensitivity, non-contact measurement, and low energy characteristics. It is sensitive to the molecular vibration, microstructure, and physical properties of substances and has shown unique advantages in food quality detection, safety screening, and component analysis. Compared to traditional methods such as near-infrared, Fourier transform infrared spectroscopy, and X-ray imaging, terahertz time-domain spectroscopy offers a complementary analytical approach with a unique perspective. While traditional infrared spectroscopy focuses on the chemical quantification of components, techniques such as X-ray imaging often suffer from insufficient contrast when examining the fine crystalline structures of light-element organic matter within rice. Terahertz waves, however, provide physical information regarding weak intermolecular interactions and low-frequency vibrations, effectively complementing the conventional methods mentioned above. Internationally, Albert Redo-Sanchez et al. verified the feasibility of THz technology in detecting antibiotic residues in food [8]. Maeng et al. achieved rapid detection of multiple pesticide residues in wheat flour using terahertz spectroscopy [9]. In China, Guo Xiangshuai analyzed the terahertz spectral characteristics of typical food additives, supporting rapid detection [10]. Wu Jingzhu et al. reviewed the applications of THz-TDS in crop and grain quality detection [11], and Li Pengpeng et al. summarized the advances of terahertz spectroscopy in harmful substance detection in food [12]. These studies confirmed the broad application potential of THz technology in food safety.

In rice-related research, Wang Qian et al. used THz-TDS combined with chemometric models to identify rice varieties with an accuracy of 95% [13]. Zhang Ying et al. realized rapid quantitative detection of pesticide residues in rice using terahertz spectroscopy [14]. Liu Yande et al. applied THz-TDS combined with machine learning to detect purple rice adulteration, in which least-squares support vector machines presented favorable performance [15].

Although previous studies have made significant progress in applying terahertz technology to rice identification, research on the classification of rice by origin and variety remains limited. The classification framework developed in this paper, which combines terahertz time-domain spectroscopy with PCA-LS-SVM, is expected to provide a feasible spectral-based reference for the preliminary screening of rice origins across different varieties.

## 2. Materials and Methods

### 2.1. Preparation of Rice Samples

**Figure 1:**
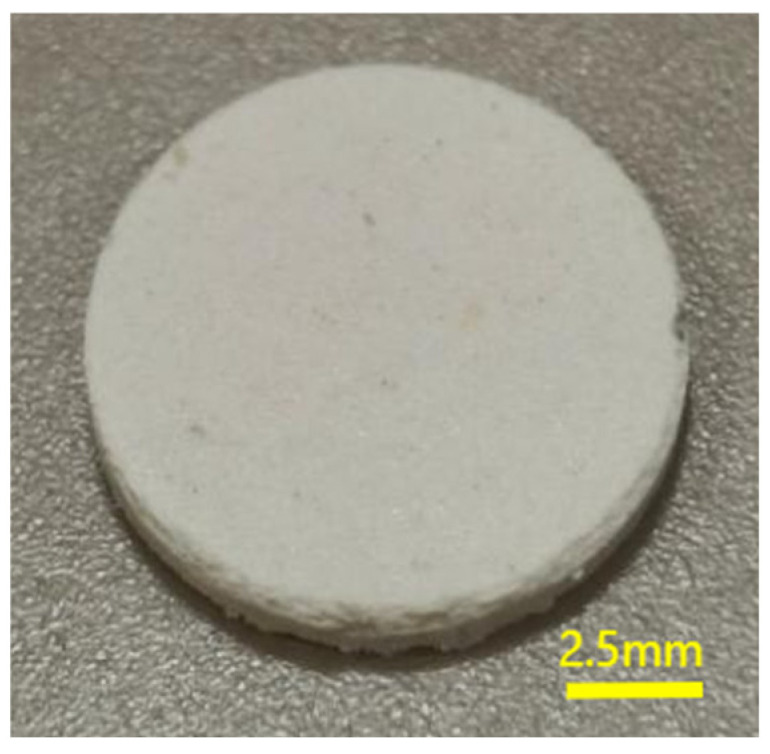
Rice tablet samples.

Four rice varieties collected from different regions were provided by the Zhejiang Academy of Agricultural Sciences. This study collected a total of 20 rice samples from different regions. To develop a classification model, four representative samples—Heilongjiang Longjing 31, Heilongjiang Jingdao No. 1, Hunan Huanghuazhan, and Hubei Ezhong No. 5 were selected from the 20 samples for algorithm optimization and modeling analysis. To eliminate the influence of extraneous variables related to temporal factors—such as variations in climatic conditions across different years—and to ensure that spectral differences stem solely from the rice’s geographical origin and varietal characteristics, the four types of rice samples used in this study were all collected during the same harvest season. Prior to sample preparation, all raw rice was properly stored in a cool, dry environment at approximately 20 °C with a relative humidity below 50%. All rice samples were dried in an oven at 80 °C for 4 h to remove moisture interference. The dried grains were ground into powder and sieved through a 200-mesh sieve to ensure uniformity. A total of 110–120 mg of rice powder was weighed using a high-precision electronic balance and then placed into a tablet press mold. The powder was compressed into circular tablets with a diameter of 10 mm and a thickness of approximately 1 mm under a pressure of 10 MPa for 50–60 s. The rice sample is shown in Figure 1. Preliminary experiments have confirmed that this thickness ensures effective penetration of terahertz waves to achieve a high signal-to-noise ratio while also producing sufficient absorption characteristics. After the pressing process was complete, the exact thickness of each tablet was measured with a vernier caliper and recorded. All prepared tablets were stored in sealed plastic bags and labeled with sample number, variety, and thickness before spectral detection. For each type of rice, 15 physically pressed samples were prepared; a total of 60 samples across the four categories were used for subsequent spectral data collection and analysis. The remaining 16 rice samples were used as a generalization test set to verify the feasibility of the final model in a multi-variety environment.

### 2.2. Extraction and Calculation of Optical Parameters

The refractive index and absorption coefficient of rice samples were calculated using the classical methods reported by Dorney et al. [16] and Duvillaret et al. [17]. First, the terahertz time-domain waveform without a sample was recorded as the reference signal. Then, the waveform transmitted through the rice sample was collected as the sample signal. Both signals were converted into the frequency domain via the Fourier transform. The refractive index and absorption coefficient were derived using the phase and amplitude information of the sample and reference signals. The relevant calculations are expressed as follows:

(1) $$n(\omega )=\frac{\varphi (\omega )c}{\omega d}+1$$

(2) $$\alpha (\omega )=\frac{2}{d}\ln\left\{\frac{4n(\omega )}{\rho (\omega ){\left[n(\omega )+1\right]}^{2}}\right\}$$

where n(ω) is the refractive index of the sample, ω is the frequency, *d* is the thickness of the sample, c is the speed of light, and φ(ω) is the phase of the ratio of the sample signal to the reference signal; where α(ω) is the absorption coefficient of the sample, and ρ(ω) is the magnitude of the ratio of the sample signal to the reference signal. These optical parameters were used as the main characteristic variables for subsequent classification and identification.

### 2.3. Spectral Data PreProcessing Methods

In terahertz detection, raw spectral data are often affected by environmental noise, moisture, mechanical vibration, and temperature–humidity fluctuations, which reduce signal-to-noise ratio and detection stability. Therefore, appropriate spectral preprocessing is essential to highlight intrinsic sample characteristics and improve modeling performance. Three widely used preprocessing methods were adopted and compared in this study. Wavelet denoising can decompose signals into multi-scale wavelet subspaces and effectively separate noise from useful information, which helps retain important feature details of the original spectra. Moving average smoothing reduces high-frequency interference by calculating the average value in a sliding window. This method is simple in calculation and shows high efficiency and stability in spectral processing. Savitzky–Golay (SG) convolution smoothing realizes noise reduction through local polynomial fitting in a sliding window. It can effectively suppress random noise while maintaining the peak shape and variation trend of the original spectra, making it widely used in terahertz spectral preprocessing [18,19,20].

### 2.4. Machine Learning Modeling Methods

Owing to the lack of obvious characteristic absorption peaks in rice terahertz spectra, machine learning methods were applied to realize accurate origin–variety identification. All modeling and data analysis were implemented in MATLAB 2020b. The following methods were used for dimensionality reduction, classification, and model evaluation.

(1) Principal Component Analysis:

PCA is an unsupervised dimensionality reduction method that converts correlated original variables into a set of linearly uncorrelated principal components (PCs) through orthogonal transformation. The first few PCs capture the majority of data variance. Generally, PCs with a cumulative variance contribution rate greater than 80% can represent most of the information of the original data. PCA was used in this study for feature extraction and data visualization [21,22]. The computational steps for PCA are as follows.

In practical applications, PCA is widely used in data preprocessing, feature extraction, image compression and other fields [23].

(2) Partial Least Squares Algorithm:

PLS-DA is a supervised classification method that integrates the advantages of PCA, canonical correlation analysis, and multiple linear regression. It extracts latent variables that maximize the covariance between spectral data and category labels, enabling effective classification even with high-dimensional and correlated variables. PLS-DA was used to establish rice origin–variety classification models [24].

(3) Least-Squares Support Vector Machines:

LS-SVM is an improved version of the standard SVM with simplified computation [25,26]. It converts quadratic programming into linear equations by using squared loss functions, thus improving calculation speed while maintaining high classification accuracy. The RBF kernel function was used in this study, which is suitable for nonlinear spectral data.

(4) BP Neural Networks:

The BP neural network is a classic feedforward network trained by error backpropagation [27]. It consists of an input layer, hidden layer(s), and output layer. Through forward calculation and backward error correction, the network continuously optimizes weights and thresholds to fit complex nonlinear relationships between spectra and categories. A single-hidden-layer BP network was constructed in this study.

**Figure 2:**
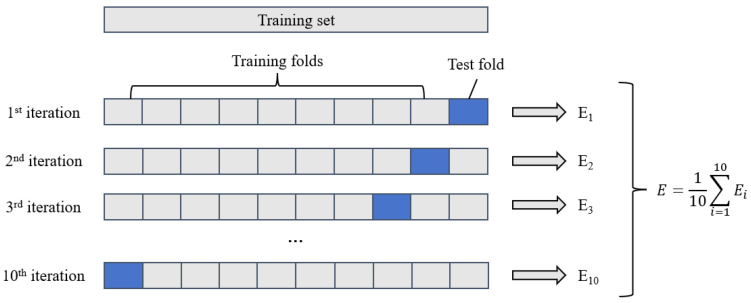
Schematic diagram illustrating the principle of K-fold cross-validation.

(5) K-fold cross-validation:

To evaluate model stability and avoid overfitting, K-fold cross-validation was used for model assessment [28]. The dataset was equally divided into K subsets. In each round, K–1 subsets were used for training and one subset for testing. This process was repeated K times, and the average accuracy was taken as the final model performance index. The principle is illustrated in Figure 2.

The results of the parameter selection for all of the above models are summarized in detail in Table 1. Throughout the entire process, the selection of parameters in Table 1 was strictly limited to the training set.

## 3. Results and Discussion

### 3.1. Spectral Acquisition and Analysis

**Figure 3:**
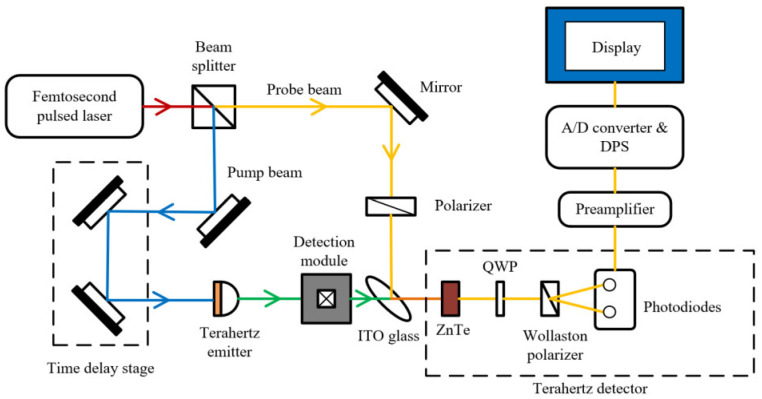
Schematic diagram of the THz-TDS system.

The terahertz spectrometer used in the experiment was purchased from Advantest Corporation, model TAS7500SP. This terahertz system has a tunable frequency range of 0.1–4 THz, a signal-to-noise ratio greater than 57 dB, and a scanning speed of less than 8 ms. Its effective spectral resolution is 7.6 GHz. The transmission-type optical path of the THz-TDS system is the most widely used; its operating principle [12] is as follows: when a femtosecond pulse enters the system, it is split by a beam splitter into two beams; one beam is stronger and serves as the pump light, whilst the other is weaker and serves as the probe light. The pump light illuminates the terahertz emitter, exciting it to generate carriers; these carriers accelerate under the influence of a bias voltage, thereby emitting a terahertz pulse. This terahertz pulse is then focused by a parabolic mirror and projected onto the sample under test. At the same time, the probe beam is focused together with the terahertz pulse onto the electro-optic zinc telluride crystal. During this process, the terahertz pulse is modulated at the sample surface due to absorption and dispersion effects, causing changes in its amplitude and phase, thereby carrying information about the sample, before continuing to be focused onto the detection crystal. After passing through a delay line, the THz pulse aligns with the detection light to jointly trigger the detector. Through the electro-optic effect, the optical signal is converted into an electrical signal, which is then processed by a phase-locked amplifier before being input into a computer for further analysis. Figure 3 shows a schematic diagram of the THz-TDS system [29].

**Figure 4:**
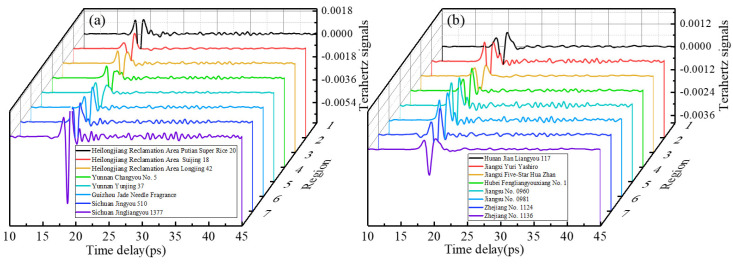
Time-domain spectra of 16 rice samples. (**a**) Time-domain plots for the first eight rice varieties; (**b**) Time-domain plots for the last eight rice varieties.

Measurements were conducted in transmission mode, with the laboratory temperature maintained at approximately 25 °C and humidity kept below 50%. Figure 4 shows the time-domain spectra of sixteen types of rice; the following section provides a detailed overview of four common types of rice, before spectral data acquisition commenced. The time-domain signals were then transformed into frequency-domain signals via a Fourier transform, and the refractive index and absorption coefficient of the thin-section samples were determined using the method proposed by Dorney, Duvillaret et al.

As the signal-to-noise ratio is lower in other frequency bands, the 0–3.0 THz range was selected as the study band for this experiment. Experimental preparations included Heilongjiang Longjing 31, Heilongjiang Jingdao No. 1, Hunan Huanghuazhan and Hubei Ezhong No. 5. Fifteen samples were prepared for each variety, totaling 60 samples. Each sample underwent five terahertz measurements, yielding a total of 300 data points, from which the mean values were calculated for plotting and analysis.

**Figure 5:**
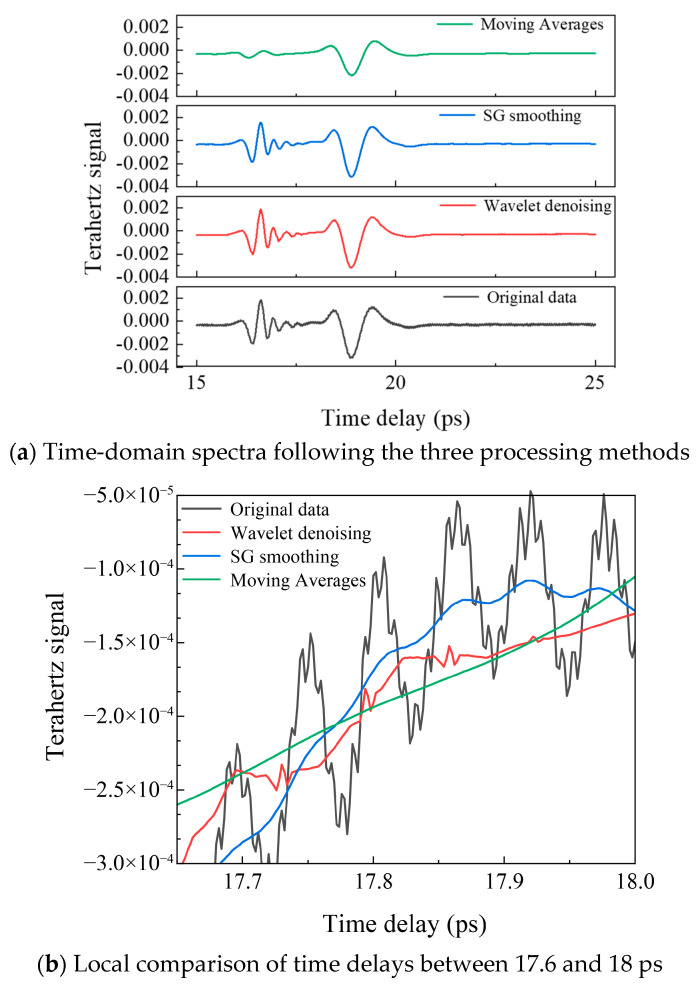
Time-domain spectrum of Heilongjiang Longjing 31.

**Figure 6:**
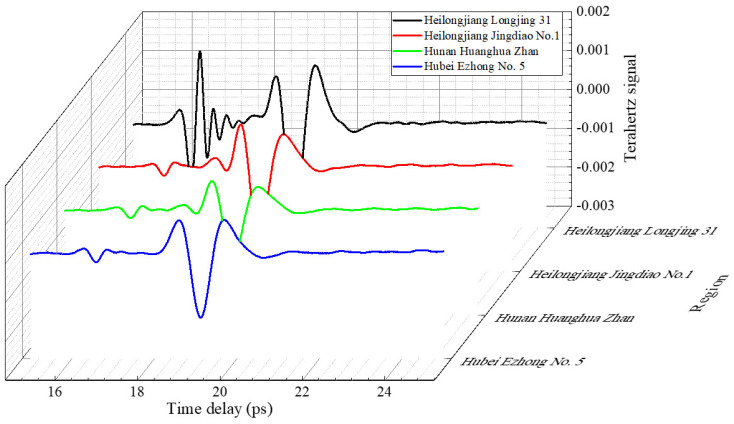
Time-domain spectra after SG smoothing.

Raw terahertz time-domain spectra were inevitably affected by environmental noise and system fluctuations. To improve the signal-to-noise ratio and enhance data smoothness, three preprocessing methods were applied, including SG convolution smoothing, wavelet denoising, and moving average smoothing. Figure 5a displays the time-domain spectra of Heilongjiang Longjing 31 after different treatments, and Figure 5b shows a local magnified comparison in the time delay range of 17.6–18 ps. Among these methods, SG smoothing achieved the best balance between noise reduction and spectral detail preservation; thus, it was adopted for subsequent spectral processing. Figure 6 illustrates the time-domain spectra of all four rice samples after SG smoothing.

**Figure 7:**
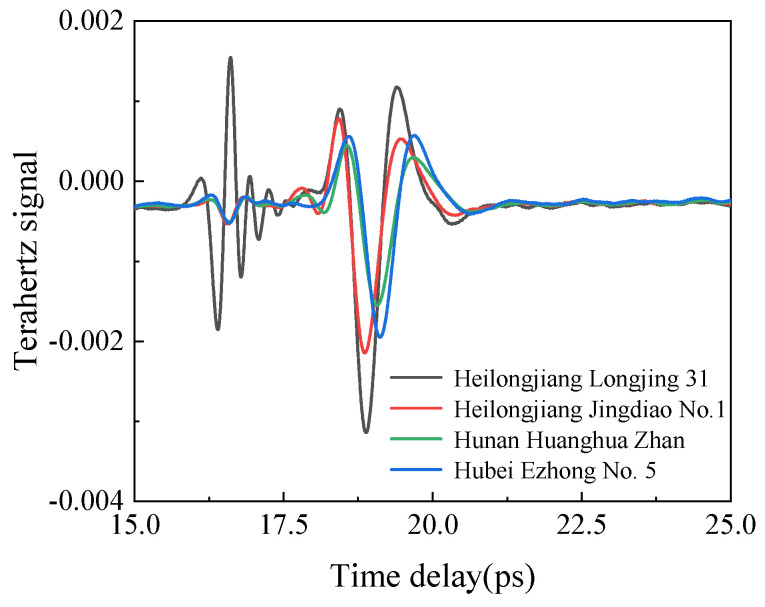
Terahertz time-domain spectra of rice samples.

**Figure 8:**
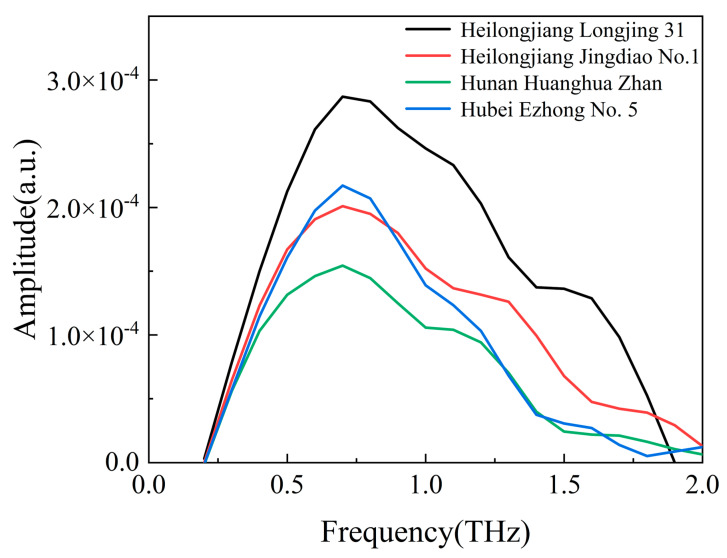
Terahertz frequency-domain spectra of rice samples.

As shown in Figure 7, the four time-domain spectra are similar, indicating that the system is very stable. Compared to the other three rice varieties, Heilongjiang Longjing 31 exhibits a larger pulse amplitude. The time delay of Heilongjiang Longjing 31 is closer to that of Heilongjiang Jingdao No. 1, while the time delays of Hunan Huanghuazhan and Hubei Ezhong No. 5 are closer to each other. Figure 8 shows the terahertz frequency-domain spectra of the four rice samples. As can be seen from the figure, all four curves exhibit a peak in the 0–2.0 THz range. The amplitude at the peak for the four different rice samples follows the relationship: Heilongjiang Longjing 31 < Hubei Ezhong No. 5 < Heilongjiang Jingdao No. 1 < Hubei Huanghuazhan. For all four rice varieties, the frequency spectrum rises first and then decreases within the 0–2.0 THz range, reaching a peak around 0.7 THz. Rice from different origins and varieties can be distinguished based on their frequency spectra, meaning that four different types of rice can be classified and identified using their frequency spectra.

**Figure 9:**
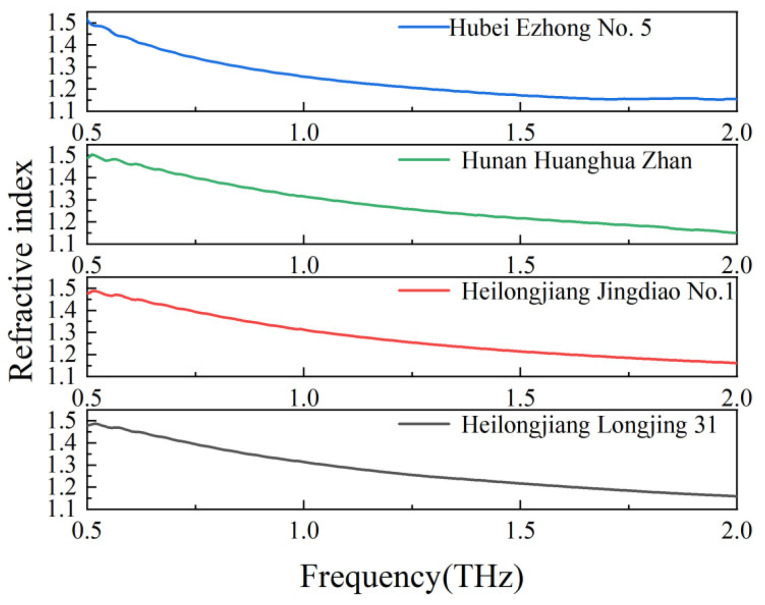
Terahertz refractive index spectra of rice samples.

Figure 9 presents the refractive index spectra of the four rice samples. Within the 0.5–2.0 THz region, the refractive index curves of Heilongjiang Longjing 31, Heilongjiang Jingdao No. 1, and Hunan Huanghuazhan were highly similar, whereas Hubei Ezhong No. 5 showed an obviously different trend. The refractive indices of all four rice varieties decreased gradually with increasing frequency, showing an inverse relationship with frequency. Such differences in refractive index reflect variations in the composition proportion and internal microstructure of rice from different producing areas. Rice from different regions varies in refractive index, which means that changes in refractive index can be used to classify and identify the origin and variety of rice.

**Figure 10:**
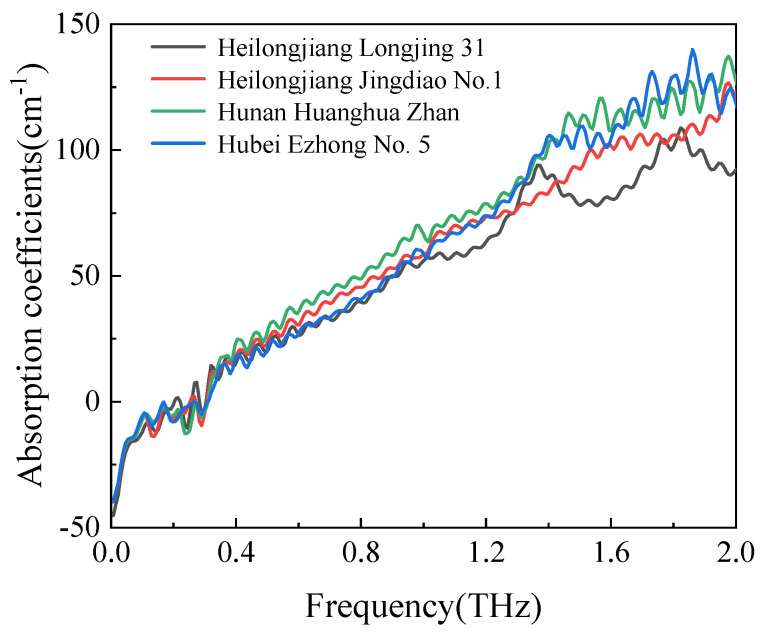
Terahertz absorption spectra of rice samples.

Figure 10 presents the absorption coefficient spectra of the four rice samples across the 0–2.0 THz range. None of the samples displayed distinct characteristic absorption peaks in this band, but the absorption coefficients of all varieties increased monotonically with rising frequency. Although the overall absorption trends of the four samples were consistent, subtle but detectable differences were observed among them. Specifically, within the high-frequency region of 1.4–2.0 THz, Heilongjiang Longjing 31 exhibited the lowest absorption coefficient, whereas Hunan Huanghuazhan and Hubei Ezhong No. 5 showed relatively higher absorption values. According to a study by Nakajima et al. [30], comparative experiments using XRD and terahertz spectroscopy confirmed that there is a very strong quantitative correlation between terahertz absorption characteristics and the crystallinity of rice starch; therefore, the characteristic differences in this wavelength band may reflect differences in the configuration of the solid macromolecular network within the rice. Further statistical analysis using one-way analysis of variance (ANOVA) revealed that the differences in the aforementioned spectral characteristics were statistically significant (*p* < 0.05) within the effective frequency range of 0.2–2.0 THz.

Amylose and amylopectin present distinct molecular and crystalline structures, leading to different collective vibrational modes. Their relative content directly determines the hardness and viscosity of cooked rice. As noted by He et al. [31] and in related studies, the multi-scale microstructure and heterogeneity of starch exert a decisive influence on its macroscopic physicochemical properties and response to physical signals. These structural differences at the microscopic level further lead to different scattering responses to terahertz radiation. Based on this theoretical framework, it can be inferred that northern japonica rice, which has a high amylopectin content, likely has a denser and more uniform internal structure, resulting in weaker scattering of terahertz waves. This may, to some extent, explain the smoother absorption curve observed in this rice; conversely, southern indica rice, which has a higher amylose content, likely exhibits a more heterogeneous microstructure, potentially leading to stronger scattering, which is consistent with the observation of more pronounced terahertz pulse broadening.

Traditionally, univariate analysis of variance (ANOVA) has been commonly used to evaluate spectral differences; however, due to the multidimensional and continuous nature of terahertz spectral data, statistics based on a single frequency point often fail to fully reflect the intrinsic differences among samples. Therefore, multivariate statistical analysis methods were employed to examine overall spectral differences. The cumulative variance explained by the first four principal components in the subsequent PCA, as well as the results of the PLS-DA model, rigorously confirmed from a multivariate statistical perspective that there are significant and distinguishable statistical differences in the spectral characteristics of rice from different origins.

Preliminary analysis indicated that the refractive index spectra of different rice samples were highly similar in profile and overlapped considerably over the whole effective frequency range. In comparison, the absorption spectra, especially in the range of 1.4–2.0 THz, showed more significant inter-class differences with distinguishable intensity variations. When the frequency exceeds 2.0 THz, high-frequency noise severely masks the sample’s useful features. Therefore, selecting the 0.2–2.0 THz range ensures that the data fed into the machine learning model possesses optimal validity and stability.

### 3.2. Dataset Partitioning Strategy

To better evaluate the true generalization capabilities of various machine learning models in classifying rice by origin and variety, this study implemented a physical sample-level data partitioning strategy using 300 spectral data points from four types of rice—representing 60 independent physical samples, each scanned five times.

As shown in Table 2, to avoid data leakage caused by partitioning samples from the same source, this study used independent physical samples rather than individual spectra as the smallest unit for dataset partitioning. For each of the 15 independent samples of each rice variety, 12 samples were randomly selected in a 4:1 ratio to construct the training set, while the remaining three samples served as an independent test set.

All five repeated spectral measurement data points from the same physical sample were fully assigned to either the training set or the test set, with no overlap between the training and evaluation procedures. All preprocessing strategies, feature extraction, and model fitting were performed exclusively on the training set, ensuring that all models were fairly compared under identical and absolutely independent testing conditions, thereby objectively reflecting the models’ true physical discrimination performance.

### 3.3. Classification and Identification Based on Principal Component Analysis

**Figure 11:**
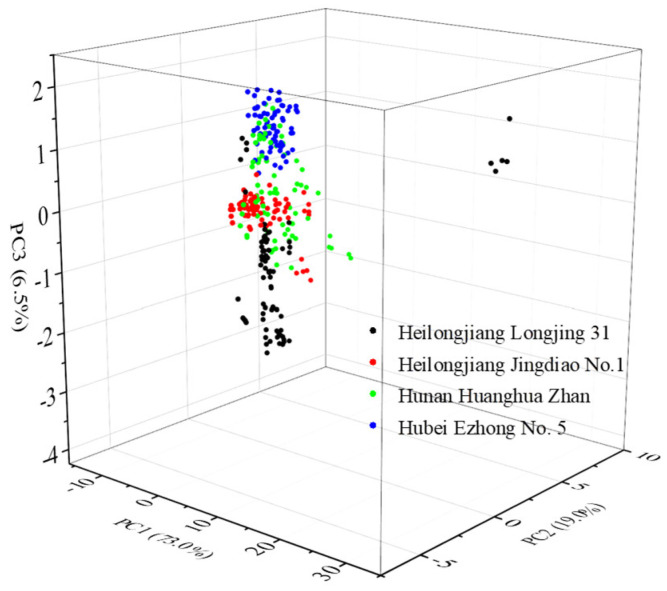
Principal component analysis scatter plot of data processed using moving average smoothing.

After applying SG convolution smoothing, wavelet denoising and moving average smoothing to the raw absorption data, the effectiveness of the principal component analysis improved following moving average smoothing. Figure 11 shows the scatter plot of the principal component analysis for the data processed with moving average smoothing. The contribution rates of the first four principal components were 73%, 19%, 6.5% and 1% respectively, with a cumulative contribution rate of 99.5%. The results of the principal component analysis indicate that this method is not effective for classifying and identifying different types of rice.

Although PCA, as a linear dimensionality reduction algorithm, successfully captured 97% of the variance in the overall data, it was unable to effectively decouple and delineate the boundaries of these nonlinear high-dimensional features, resulting in severe overlap among different varieties in the low-dimensional projection space. This further underscores the necessity of subsequently introducing LS-SVM, which possesses strong nonlinear mapping capabilities, for classification modeling.

### 3.4. Classification and Identification Based on the Partial Least Squares Discrimination Algorithm

In the partial least squares classification, the true values 1–4 were assigned to represent Heilongjiang Longjing 31, Heilongjiang Jingdao No. 1, Hunan Huanghuazhan and Hubei Ezhong No. 5, respectively.

**Figure 12:**
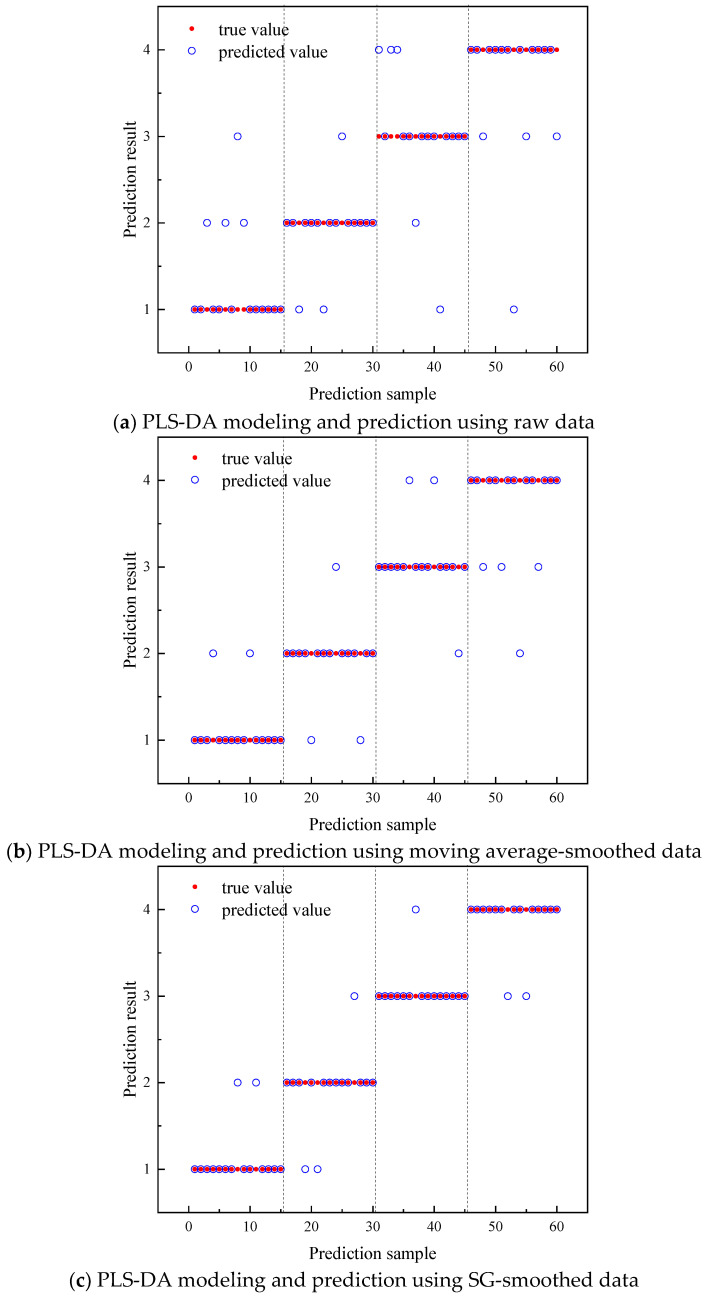
PLS-DA modeling predictions.

The results of the raw data modeling based on partial least squares discriminant analysis are shown in Figure 12a. There were 16 misclassifications in the prediction samples, and the overall classification accuracy was 73.3%. To optimize model performance, this study compared two classical spectral preprocessing methods. As shown in Figure 12b, the use of moving average smoothing increased the overall accuracy to 80%. However, as seen in Figure 12c, the application of the SG convolution smoothing algorithm resulted in a breakthrough improvement in model performance. This method reduced the number of misclassified samples to eight, with the overall accuracy reaching 86.6%. Compared to the raw data and moving average processing, the SG algorithm achieved accuracy improvements of 13.3% and 6.6%, respectively, demonstrating significant optimization effects. A comprehensive comparison shows that the best results for PLS-DA are achieved using data processed with SG smoothing. The model’s stability was evaluated using 5-fold cross-validation. Following the dataset partitioning strategy described in Section 3.2, all five replicate spectral measurements from the same physical tablet were assigned entirely to either the training set or the test set, with no overlap between the training and evaluation sets. The final average accuracy of the 5-fold cross-validation was 85.3%.

### 3.5. Classification and Recognition Based on Partial Least-Squares Support Vector Machines

In the classification and recognition using LS-SVM, the true values 1–4 were assigned to represent Heilongjiang Longjing 31, Heilongjiang Jingdao No. 1, Hunan Huanghuazhan and Hubei Ezhong No. 5, respectively. The RBF_kernel function within the LS-SVM module is used to model the raw data, as well as the data processed using moving average smoothing and SG smoothing, for the rice varieties.

**Figure 13:**
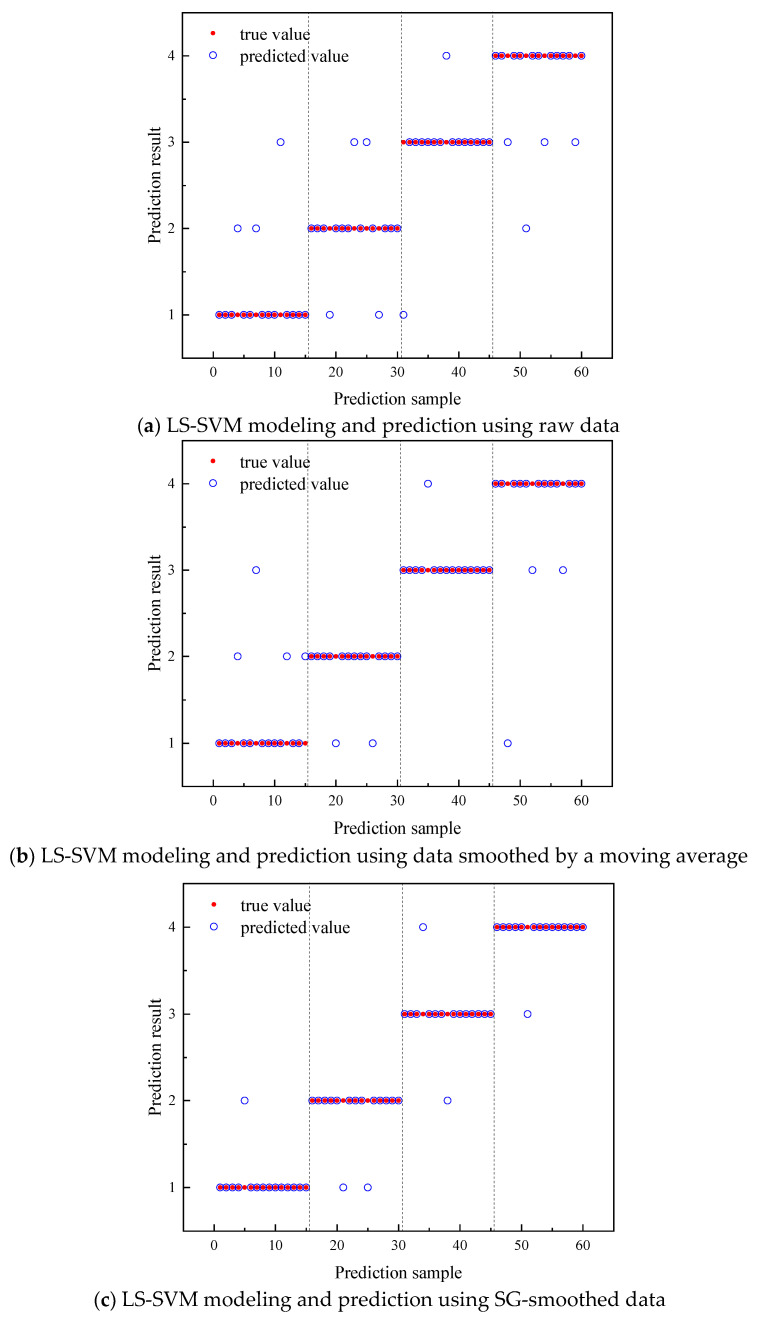
LS-SVM modeling predictions.

By comparing the prediction results of LS-SVM modeling using raw data, data smoothed by moving average, and data smoothed by SG, as shown in Figure 13a, it can be seen that the predictions based on raw data resulted in 13 misclassifications, with an accuracy rate of 78.3%; however, as shown in Figure 13b, after moving average smoothing, the number of misclassifications was reduced to 10, and the accuracy rate increased to 83.3%. Furthermore, as shown in Figure 13c, after applying SG smoothing, there were only six misclassifications, and the accuracy rate reached 90%. This indicates that both moving average and SG smoothing can effectively reduce data noise and optimize model inputs, thereby improving prediction accuracy. Among these, SG smoothing demonstrates more significant effects, and the SG smoothing algorithm provides a feasible reference for the subsequent development of a rice category model. Using the same method as in the previous section, we evaluated the model’s stability through 5-fold cross-validation, which yielded an average accuracy of 88.6%.

### 3.6. Classification and Recognition Based on BP Neural Networks

**Figure 14:**
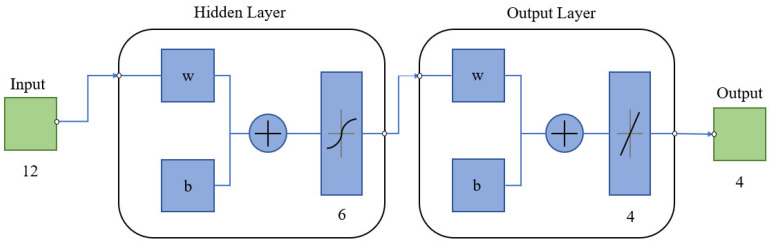
Pre-established BP neural network topology.

**Figure 15:**
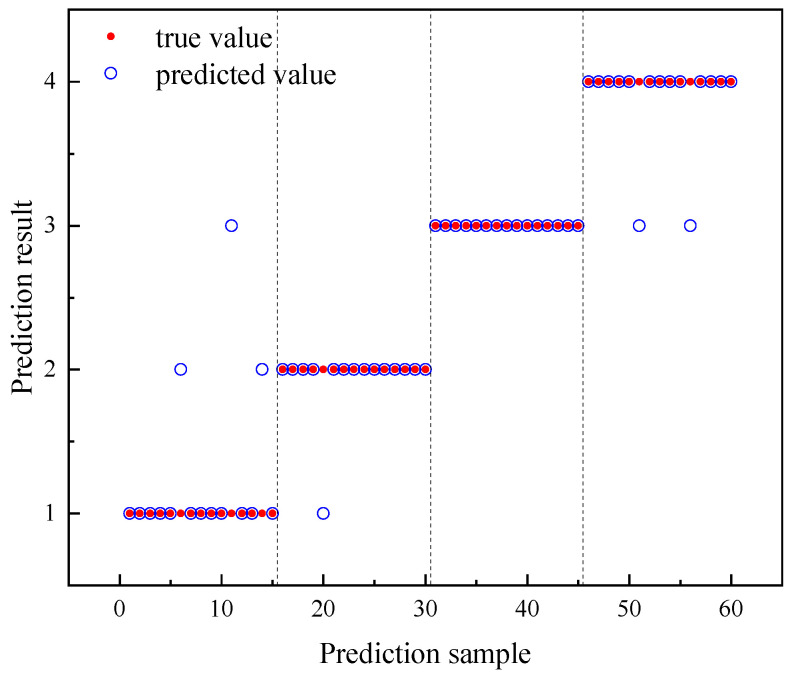
Prediction results from the BP neural network model.

In a BP neural network, the appropriate selection of parameters has a significant impact on model performance. Given that a shallow BP neural network containing only one hidden layer is already capable of realizing any n-to-m dimensional mapping within a closed interval [32], this study constructed a BP neural network model with a single hidden layer. To further improve network performance, 12 effective features were extracted from each group within the 0.2–2.0 THz range. Through systematic adjustment of key parameters such as the number of neurons, learning rate and number of iterations, a set of parameter configurations was ultimately determined that performed excellently in terms of both accuracy and efficiency: six neurons in the hidden layer, a learning rate of 0.01, and a maximum of 180 iterations. The model’s output layer comprises four nodes, corresponding respectively to the four different rice varieties, and its network structure is shown in Figure 14. Based on the pre-defined BP neural network model, the true values 1–4 were assigned to represent Heilongjiang Longjing 31, Heilongjiang Jingdao No. 1, Hunan Huanghuazhan and Hubei Ezhong No. 5. Figure 15 shows that the prediction accuracy reached 90%. Experiments demonstrate that the proposed parameter optimization method not only improves the model’s recognition accuracy but also enhances its applicability in complex real-world scenarios.

### 3.7. Discriminant Analysis Using PLS-DA and LS-SVM Based on PCA Scores

To establish a classification model for high-dimensional data, this study adopted a strategy combining principal component analysis with PLS-DA. PCA was used to identify the feature variables contributing most significantly to data variability, thereby constructing a classification model with good generalization capability. The selection of spectral preprocessing strategies, the calculation of the covariance matrix for PCA, and the fitting of eigenvectors are all performed on the training set. All feature variables were normalized to eliminate the influence of dimensionality differences on the model.

**Figure 16:**
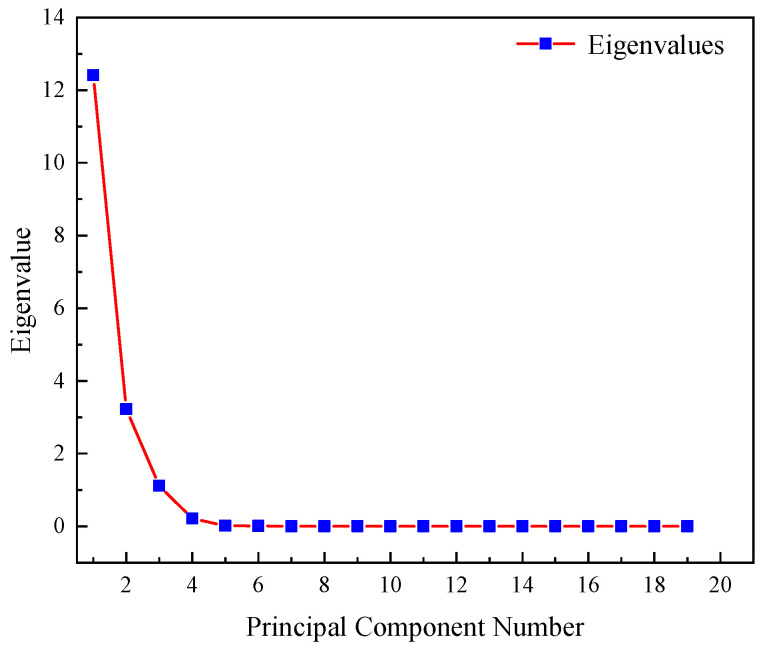
Principal component analysis scatter plot.

First, unsupervised principal component analysis was performed on the pre-processed absorption spectrum data matrix, as shown in Section 3.3, to reduce the data dimension and eliminate multicollinearity. PCA transforms the original features into a series of uncorrelated principal components via linear transformation, where the first principal component contains the maximum variance of the data, and subsequent principal components sequentially contain the remaining variance. Subsequently, by examining the scree plot in Figure 16, it was found that the sum of the eigenvalues of the first four principal components is 16.966, and the cumulative contribution rate of the first four principal components is defined as the ratio of the sum of their eigenvalues to the sum of all eigenvalues. Therefore, the scores of the first four principal components, which account for over 99% of the cumulative contribution, were selected as new predictor variables. These were then incorporated, together with the sample class labels, into a supervised partial least squares discriminant analysis (PLS-DA) to construct a classification model. This strategy effectively preserves the primary variation in the data whilst significantly reducing the feature dimension, thereby helping to improve the model’s computational efficiency and generalization ability.

**Figure 17:**
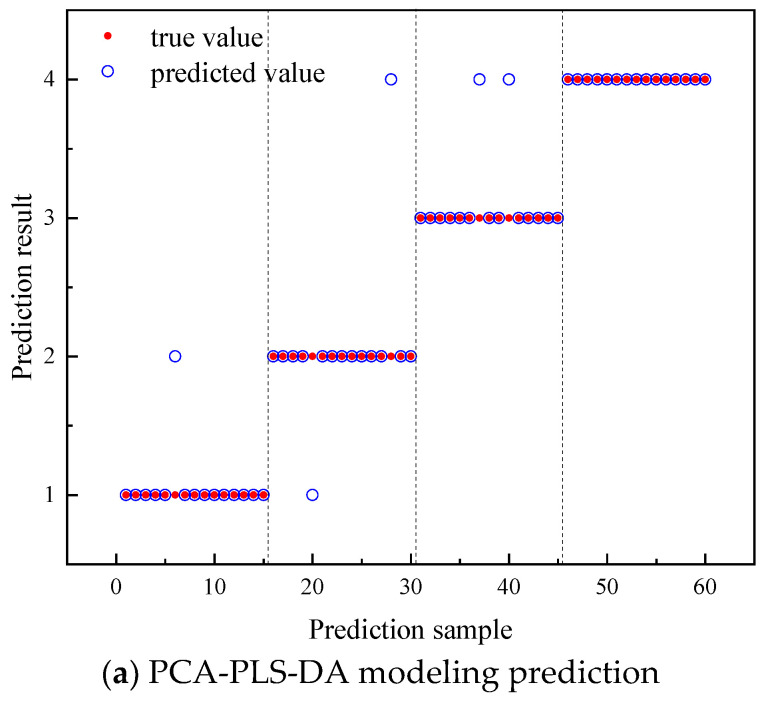

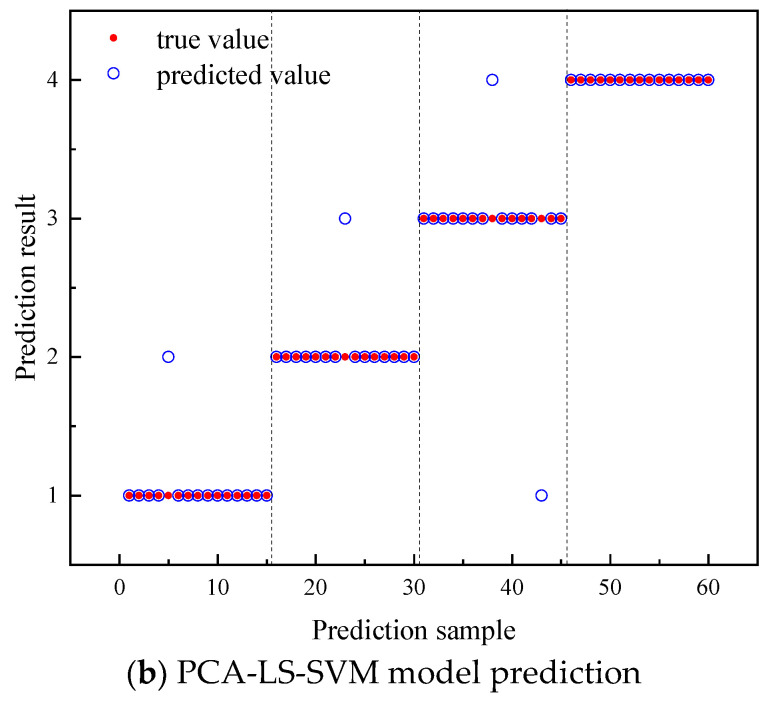
Prediction based on PCA score inputs.

**Figure 18:**
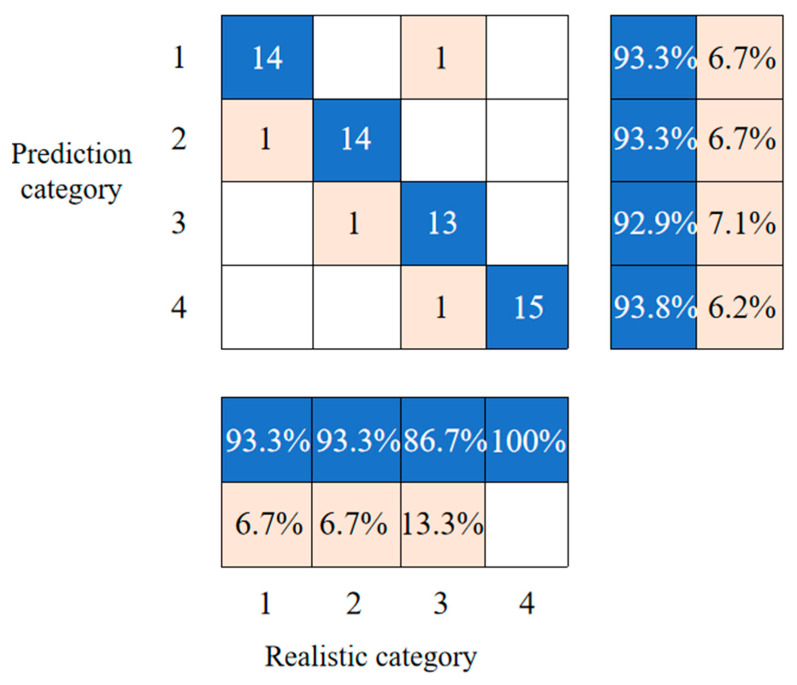
Confusion matrix for PCA-LS-SVM modeling and prediction.

PCA-PLS-DA and PCA-LS-SVM models were constructed, with true values 1–4 representing Heilongjiang Longjing 31, Heilongjiang Jingdao No. 1, Hunan Huanghuazhan, and Hubei Ezhong No. 5, respectively. The prediction results are shown in Figure 17 below. The PCA-PLS-DA model had five prediction errors, achieving an accuracy rate of 91.6%, while the PCA-LS-SVM model had only four prediction errors, achieving an accuracy rate of 93.3%. To evaluate the models’ generalization performance and reduce overfitting, 5-fold cross-validation was employed, resulting in final average cross-validation accuracy rates of 89.6% and 91.3%, respectively. The prediction scatter plots shown in Figure 17b visually demonstrate the models’ refined identification performance across specific categories. The precision and recall rates for different types of rice are shown in Figure 18. The optimized model achieved an overall F1 score of 93.27% on the test set. This method of direct graphical mapping not only clearly identifies the specific categories where misclassifications occur but also effectively avoids redundant data presentation.

### 3.8. Comparative Analysis of Models

The results indicate that, when dealing with spectral data characterized by nonlinear and complex interactions, unsupervised dimension reduction methods must be combined with supervised learning algorithms to achieve maximum effectiveness. Among supervised models, PLS-DA and LS-SVM performed exceptionally well in classifying different rice varieties. Specifically, SG-smoothed PLS-DA achieved an accuracy of 86.6%, while SG-smoothed LS-SVM performed even better, reaching 90% accuracy. Subsequently, absorption coefficients were used for feature extraction to enable classification prediction via a backpropagation (BP) neural network, which also achieved 90% accuracy. Finally, a discriminant analysis was conducted using PCA scores as input for PLS-DA and LS-SVM. The PCA-PLS-DA model achieved an accuracy of 91.6%, while the PCA-LS-SVM model had only four misclassifications, resulting in an accuracy of 93.3%. Therefore, the combination of PCA-LS-SVM with terahertz time-domain spectroscopy technology enables the identification and detection of rice from different origin–variety combinations.

### 3.9. Evaluating the Generalization Capabilities of Classification Models in Low-Sample-Size Scenarios

Based on samples from four typical production regions, a PCA-LS-SVM classification and detection model was systematically constructed and validated. In this phase, to further verify the generalization capability of the optimal analytical framework, this study introduced an additional 16 rice samples. Only three independent pressings were collected for each rice variety, resulting in a total of 48 micro-samples. Given the extremely small sample size, using a conventional fixed-ratio split would likely lead to distorted model evaluation. Therefore, this study combined these 16 newly added rice varieties with the 60 pressings from the four typical rice varieties mentioned earlier to construct a qualitative classification framework for 20 rice varieties. The 60 samples from the previous study served as a resident background knowledge base to solidify the mathematical framework of the classifier. An independent leave-one-out cross-validation experiment was conducted on the 48 newly added samples to ensure that each of the 48 samples was predicted once as an independent test set, thereby maximizing the utilization of limited data and objectively evaluating the model’s true generalization capability. All raw spectra underwent the optimal feature extraction workflow established in the previous study, specifically, SG smoothing followed by the extraction of principal component scores for input into the PCA-LS-SVM model. The cross-validation results indicate that even under stringent conditions where only a very small number of samples from each class were used for training, the PCA-LS-SVM model successfully identified 43 out of the 48 independent test samples, achieving an overall prediction accuracy of 89.6%.

A further analysis of the distribution characteristics of the five misclassified samples in Table 3 reveals that two of the misclassifications occurred between Jiangsu 0960 and Jiangsu 0981, while the other two involved the Sichuan Jingyou 510 sample being misclassified as Sichuan Jingliangyou 1377. Heilongjiang Nongken Putian Super Rice 20 was misclassified as Heilongjiang Nongken Longjing 42, and only one instance involved Zhejiang No. 1124 being misclassified as Jiangsu No. 0960 from another province. These instances of misclassification were all confined to the same province or to adjacent geographical locations in neighboring provinces.

This expanded sample test indicates that, under conditions of limited sample size, the model may still produce classification errors for varieties from the same province or those located in close proximity with similar climatic conditions. Nevertheless, the combination of terahertz time-domain spectroscopy and the PCA-LS-SVM architecture demonstrated stability even with limited data resources. Under controlled laboratory conditions, this study provides preliminary evidence of the feasibility of applying this technology to the classification of agricultural products by origin.

## 4. Conclusions

This study used four types of rice from different production regions as experimental materials to investigate the feasibility of combining terahertz time-domain spectroscopy with machine learning modeling for the classification of rice by origin and variety. The study also completed the collection, preprocessing, and feature extraction of spectral data, as well as the development of classification models.

(1) The optical parameters, such as refractive index and absorption coefficient of rice samples, were obtained by THz-TDS, and SG convolution smoothing, wavelet denoising and moving average smoothing were used for spectral preprocessing. The results indicate that rice from different origins and varieties exhibits significant differences in its terahertz spectral characteristics, which can be utilized for the classification and identification of rice.

(2) PCA, PLS-DA, LS-SVM and BP neural network were used to establish classification models. PCA-PLS-DA and PCA-LS-SVM models have excellent performance, with classification accuracy of 91.6% and 93.3% respectively.

(3) The combination of terahertz time-domain spectroscopy and machine learning enables relatively accurate identification of rice origin and variety. This study validated the feasibility of this technical framework for rice origin and variety classification.

(4) Based on the four sample types, we successfully expanded the classification scope to 20 rice varieties nationwide and preliminarily confirmed the stability of the PCA-LS-SVM model; however, the absolute volume of data from the expanded samples remains limited. The terahertz spectral characteristics of rice exhibit complex variations driven by geographical microclimates and the duration of storage. Future work will focus on collaborating with producers across multiple regions to construct a standard terahertz spectral database for rice under complex storage conditions, thereby advancing the application of non-destructive category.

## Figures and Tables

**Table 1:** Summary of preprocessing and model parameter selection.

Pipeline Stage	Parameter/Setting	Final Selected Value
SG Smoothing	Window Length	31
	Polynomial Order	3
Moving Average	Window Size	21
Wavelet	Wavelet Family	db4
	Decomposition Level	6
PCA	Number of Principal Components (PCs)	4
LS-SVM	Kernel Function	RBF
	Regularization Parameter	36.5958
	Kernel Parameter	1.4392

**Table 2:** Dataset splits for all machine learning models.

Model	Number of Physical Pellets	Number of Spectra	Train/Test Split (Pellet Level)	Train/Test Split (Spectrum Level)	Repeated Measurements Treatment	Spectra from Same Pellet in Both Sets?
All model	60	300	48 Train /12 Test	240 Train /60 Test	Used separately	No

**Table 3:** Records of misclassification in the classification prediction of 16 rice samples.

Actual Sample Type	Number of Misjudged Clips	Types of Misjudgments
Jianesu No. 0960	1	Jiangsu No. 0981
Jiangsu No. 0981	1	Jianesu No. 0960
Sichuan Jingyou 510	1	Sichuan Jingliangyou 1377
Heilongjiang Reclamation Area Putian Super Rice 20	1	Heilongjiang Reclamation Area Longjing 42
Zhejiang No. 1124	1	Jianesu No. 0960

## Data Availability

The original contributions presented in this study are included in the article. Further inquiries can be directed to the corresponding authors.
